# Handling method affects measures of anxiety, but not chronic stress in mice

**DOI:** 10.1038/s41598-022-25090-9

**Published:** 2022-12-03

**Authors:** Janja Novak, Ivana Jaric, Marianna Rosso, Reto Rufener, Chadi Touma, Hanno Würbel

**Affiliations:** 1 Animal Welfare Division, Vetsuisse Faculty, University of Bern, Bern, Switzerland; 2 Department of Infectious Diseases and Pathobiology, Vetsuisse Faculty, Institute of Parasitology, University of Bern, Bern, Switzerland; 3 Department of Behavioural Biology, Osnabrück University, Osnabrück, Germany

**Keywords:** Animal behaviour, Animal physiology

## Abstract

Studies in mice have shown that less aversive handling methods (e.g. tunnel or cup handling) can reduce behavioural measures of anxiety in comparison to picking mice up by their tail. Despite such evidence, tail handling continues to be used routinely. Besides resistance to change accustomed procedures, this may also be due to the fact that current evidence in support of less aversive handling is mostly restricted to effects of extensive daily handling, which may not apply to routine husbandry practices. The aim of our study was to assess whether, and to what extent, different handling methods during routine husbandry induce differences in behavioural and physiological measures of stress in laboratory mice. To put the effects of handling method in perspective with chronic stress, we compared handling methods to a validated paradigm of unpredictable chronic mild stress (UCMS). We housed mice of two strains (Balb/c and C57BL/6) and both sexes either under standard laboratory conditions (CTRL) or under UCMS. Half of the animals from each housing condition were tail handled and half were tunnel handled twice per week, once during a cage change and once for a routine health check. We found strain dependent effects of handling method on behavioural measures of anxiety: tunnel handled Balb/c mice interacted with the handler more than tail handled conspecifics, and tunnel handled CTRL mice showed increased open arm exploration in the elevated plus-maze. Mice undergoing UCMS showed increased plasma corticosterone levels and reduced sucrose preference. However, we found no effect of handling method on these stress-associated measures. Our results therefore indicate that routine tail handling can affect behavioural measures of anxiety, but may not be a significant source of chronic husbandry stress. Our results also highlight strain dependent responses to handling methods.

## Introduction

The laboratory environment, including housing conditions and routine handling procedures can be a source of stress to laboratory animals and alter their physiological and behavioural responses to experimental treatments^1–5^. For example, an active transfer of mice to a cage leads to a higher increase in plasma corticosterone levels compared to a passive transfer without handling^6^. By altering behaviour and physiology, handling might thus affect not only animal welfare, but also the quality of experimental data, contributing to unexplained variation in research outcomes. Effects of handling on behaviour and physiology are complex and may depend on several factors, such as genotype^7^, the rearing environment^3,8,9^, and the extent of handling^10^. Some studies suggest however, that the standard handling method of picking mice up by their tail increases behavioural and physiological measures of stress (e.g. behavioural measures of anxiety^11–15^, anhedonia^14^, and despair^16^, altered hypothalamic–pituitary–adrenal (HPA) axis activation^17^, and breeding productivity^18^) compared to less aversive handling methods (e.g. tunnel or cup handling).

Despite some evidence indicating that tail handling may be more stressful for mice than less aversive handling methods, tail handling continues to be used routinely^19^. However, according to a survey by Henderson et al.^19^, further evidence is needed, particularly for representative real-life scenarios in biomedical research (e.g. evidence of handling impact on stress physiology), which would increase the likelihood of switching to less aversive handling methods. Current evidence in support of cup or tunnel handling is mostly limited to behavioural outcomes. Most consistently, it was found that tail handling reduced voluntary interaction of the mice with the experimenter compared to tunnel handling^12,14,15^. Effects of handling method on other markers of stress have been less consistent across different strains and both sexes^11,13,16,20–22^. Another argument against wider adoption of less aversive handling methods is duration and frequency of handling^19^. Adverse effects of tail handling have mostly been demonstrated after extensive daily handling (i.e. holding mice by their tails for 30 s, twice a day, for 7–10 consecutive days)^12–14,17^, which, however, does not apply to routine husbandry practices with brief handling episodes once or twice per week during cage changes and other routine procedures. So far, only a few studies showed that even brief tail handling (2s) can affect behaviour^12,23^.

Some of the reported effects of tail handling are strikingly similar to those induced by unpredictable chronic mild stress (UCMS), a paradigm commonly used with laboratory mice to induce and study chronic mild stress^24–26^. The paradigm involves exposing the animals to mild unpredictable stressors over several weeks, which results in behavioural, neuroendocrine and morphological changes (e.g. behavioural measures of anxiety, increased HPA axis activity and reactivity to acute stressors, anhedonia, adrenal gland hypertrophy and thymus atrophy)^24,27–36^. Since handling is a necessary component of animal husbandry, reducing handling stress can have an immense effect on both the welfare of laboratory mice and the quality of research data.

The aim of this study was to assess whether, and to what extent, tail handling during routine husbandry induces stress-related behavioural and physiological changes in laboratory mice, and whether tunnel handling can attenuate these effects. To this end, we first assessed whether we could replicate the effects of tail handling compared to tunnel handling using the original extensive handling protocols (Experiment 1). In a second experiment, we compared the effects of tail and tunnel handling when using a routine husbandry protocol of brief handling twice weekly for health checks and cage changes. To put the effects of tail handling in perspective with other forms of chronic stress, we compared these effects to those of a validated paradigm of UCMS^24^. If routine tail handling acts as a chronic stressor in laboratory mice, we hypothesized that tail handled mice would display similar phenotypic changes to mice undergoing UCMS.

## Results

### Experiment 1: Daily handling for nine consecutive days

During the nine days of handling, aggression escalated in four cages of male mice (three Balb/c and one C57BL/6). The animals in these cages were housed singly until the end of the experiment. One C57BL/6 male mouse had to be euthanized before testing due to wounding. Further, seven voluntary interaction trials had to be interrupted because of home cage aggression during the trial, so these trials were not included in the analysis. Complete results from the full model run are presented in Table 1.

#### Voluntary interaction test (VIT)

**Figure 1:**
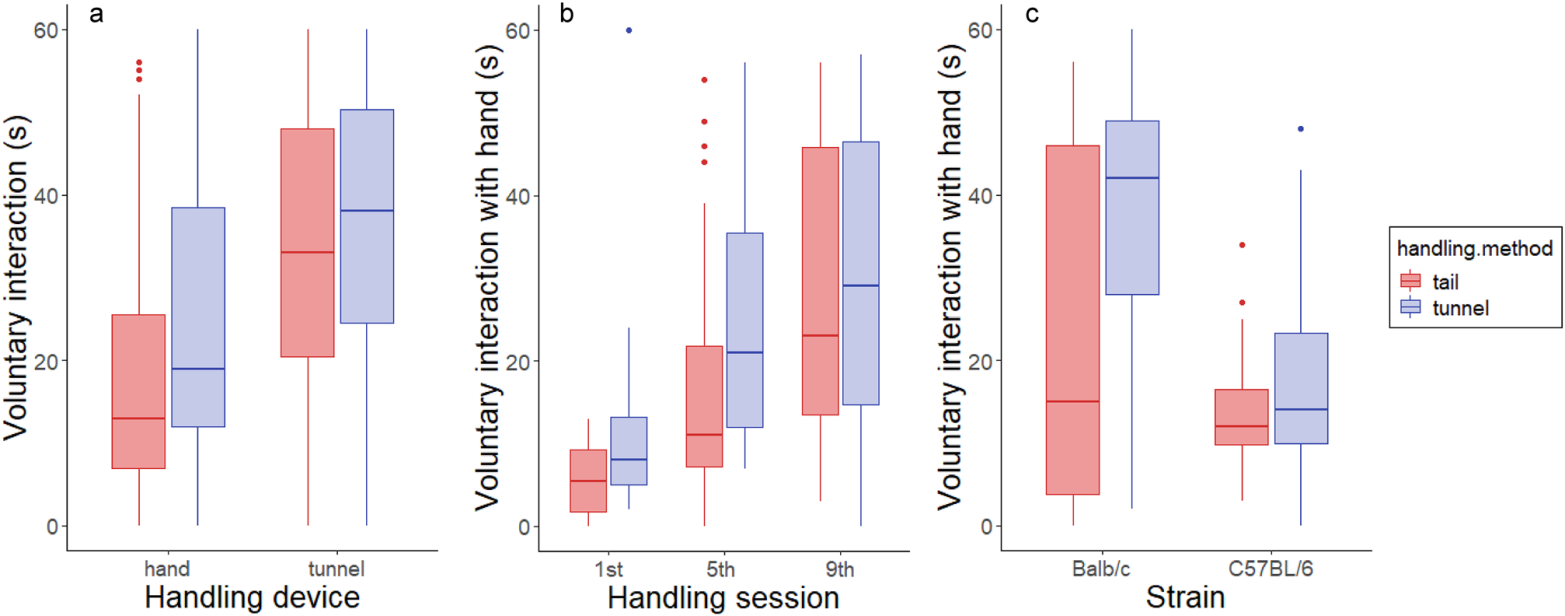
Voluntary interaction with both handling devices, experimenter's hand and tunnel, when mice were handled daily for nine consecutive days. (**a**) Mice interacted with the tunnel more than with the experimenter's hand. (**b**) Voluntary interaction with the experimenter's hand increased across the three handling sessions for all mice. (**c**) Balb/c mice interacted more with the experimenter's hand compared to C57BL/6 mice. Here, both interaction before and after handling are included.

Time spent in voluntary interaction with the experimenter was influenced by the handling device with which interaction was measured. All mice spent more time interacting with the tunnel than with the hand (F_1,279_ = 95.73, *P* < 0.001; Fig. 1a). Therefore, subsequent analyses of the VIT were done on time spent interacting with the hand. Overall, mice spent 35 ± 1 s interacting with the tunnel and 22 ± 1 s with the hand (mean ± SE).

Handling method did not have a strong effect on voluntary interaction with the experimenter's hand (F_1,10_ = 2.23, *P* = 0.33), although tunnel handled Balb/c mice seemed to have increased interaction compared to tail handled mice (Fig. 1c). Time spent interacting with the hand before and immediately after handling was also not different (F_1,137_ = 3.15, *P* = 0.16). However, interaction time increased across the three handling sessions for all mice, from 14 ± 2 s to 34 ± 2 s (F_2,131_ = 20.58, *P* < 0.001; Fig. 1b). Balb/c mice spent more time interacting with the hand than C57BL/6 mice (29 ± 3 s compared to 16 ± 1 s; F_1,10_ = 9.30, *P* = 0.02).

#### Elevated plus-maze (EPM)

**Figure 2:**
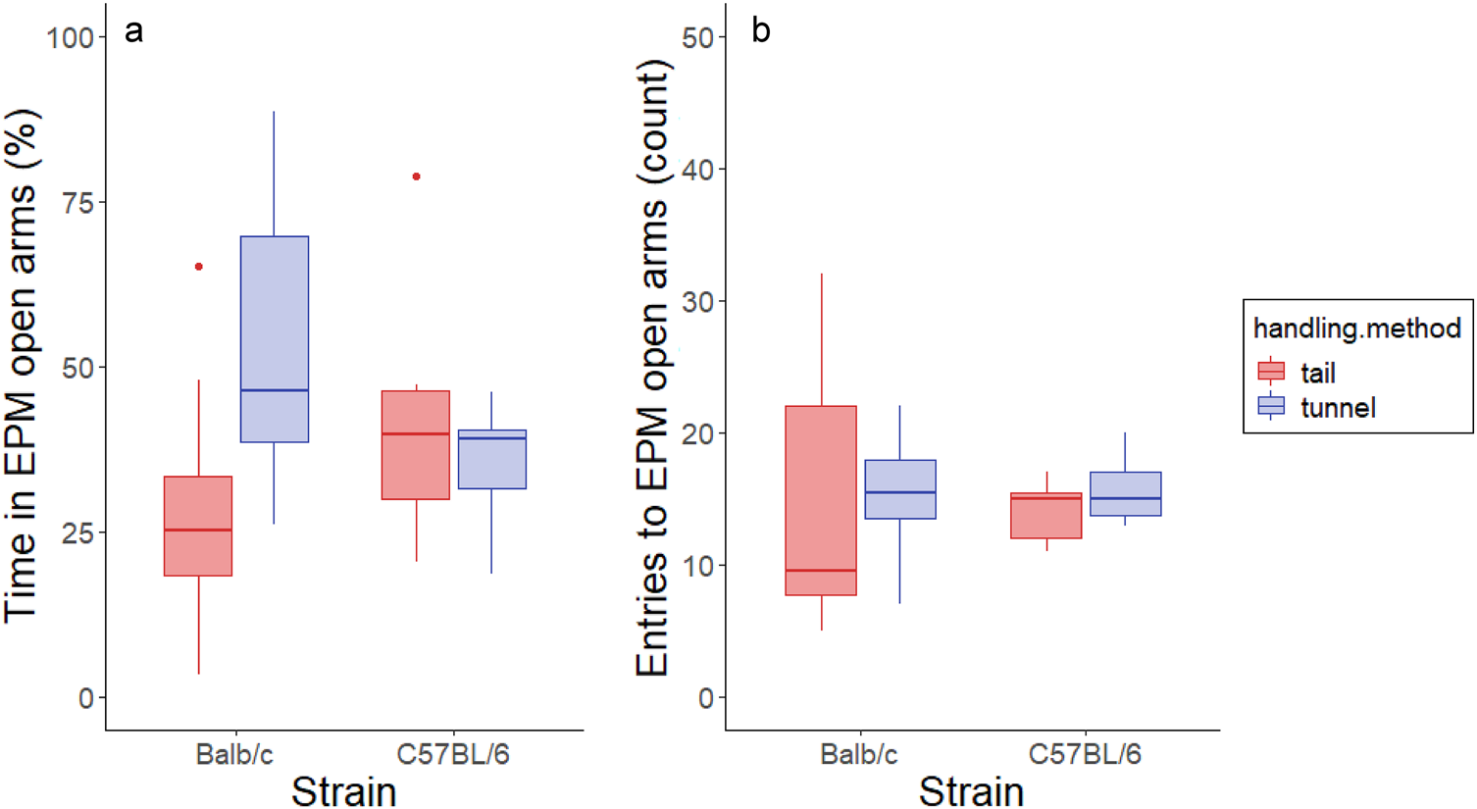
Behaviour on the elevated plus-maze. (**a**) Tail handled Balb/c mice spent less time in open arms of the EPM compared to tunnel handled mice while there was no effect of handling method in C57BL/6 mice. (**b**) Handling method had no effect on the number of open arm entries.

Handling method affected time spent in the open arms of the EPM, but only in Balb/c mice (handling method × strain F_1,25_ = 5.21, *P* = 0.03; Fig. 2a). Tunnel handled Balb/c mice spent almost twice as much time in the open arms as tail handled mice (53 ± 8% compared to 29 ± 7%). In C57BL/6 mice, time spent in open arms was similar for both handling groups (36 ± 3 and 42 ± 7% for tunnel and tail handled mice, respectively). However, there was no strong effect of handling method on the number of entries to open arms (F_1,25_ = 0.26, *P* = 0.62; Fig. 2b), and handling method had no influence on total distance travelled in the EPM (F_1,25_ = 1.18, *P* = 0.29).

#### Thymus and adrenal gland weights

Thymus and adrenal gland weights did not differ between tunnel or tail handled mice (thymus; F_1,24_ = 0.09, *P* = 0.77, adrenal glands; F_1,24_ = 0.80, *P* = 0.39). Females had larger adrenal glands (F_1,24_ = 0.86.85, *P* < 0.001) and thymus (F_1,24_ = 13.91, *P* = 0.004) than males. Relative thymus weight also differed between strains, with C57BL/6 mice having heavier thymus (F_1,24_ = 12.05, *P* = 0.006, Figure S1). Handling method affected body weight at euthanasia, with tunnel handled males being heavier than tail handled males (F_1,8_ = 7.38, *P* = 0.03).

### Experiment 2: Weekly handling and UCMS

15 of 640 voluntary interaction trials were excluded from the analysis either because of a mouse biting the handler's hand so the trial was discontinued or due to an error made during testing (wrong order of handling devices). Two mice were excluded from the nest score analysis because of an error made with nesting material. One mouse was euthanized before the HPA reactivity test due to wounding. Complete results from the full model run are presented in Table 2.

**Table 1:** Test statistics for the full models run in Experiment 1, where mice were handled daily for nine days. Handling method, strain and sex were included as fixed factors and home cage nested in experimenter was included as a random factor.

Experiment 1	Fixed factors								
Outcome measures	Handling method	Strain	Sex	Handling method × strain	Handling method × sex	Before/after handling	Handling session	Handling method × before/after	Handling method × session
Voluntary interaction with hand	F_1,10_ = 2.23 *P* = 0.33	**F**_**1,10**_** = 9.30** ***P* = 0.02**	F_1,10_ = 0.002 *P* = 0.97	F_1,10_ = 1.08 *P* = 0.64	F_1,10_ = 0.67 *P* = 0.86	F_1,137_ = 3.15 *P* = 0.16	**F**_**1,137**_** = 20.58** ***P* < 0.001**	F_1,131_ = 0.004 *P* = 0.95	F_1,135_ = 0.003 *P* = 0.95
Time in EPM open arms	F_1,25_ = 2.47 *P* = 0.13	F_1,25_ = 0.001 *P* = 0.97	F_1,25_ = 0.19 *P* = 0.67	**F**_**1,25**_** = 5.21** ***P* = 0.03**	F_1,25_ = 0.53 *P* = 0.48	–	–	–	–
Entries to EPM open arms	F_1,25_ = 0.26 *P* = 0.62	F_1,25_ = 0.005 *P* = 0.94	F_1,25_ = 0.57 *P* = 0.47	F_1,25_ = 0.005 *P* = 0.94	F_1,25_ = 0.07 *P* = 0.79	–	–	–	–
Adrenal gland weight	F_1,24_ = 0.80 *P* = 0.39	F_1,24_ = 4.49 *P* = 0.06	**F**_**1,24**_** = 86.85** ***P* < 0.001**	F_1,24_ = 0.21 *P* = 0.65	F_1,24_ = 0.10 *P* = 0.76	–	–	–	–
Thymus weight	F_1,24_ = 0.09 *P* = 0.77	**F**_**1,24**_** = 12.05** ***P* = 0.006**	**F**_**1,24**_** = 13.91** ***P* = 0.004**	F_1,24_ = 0.74 *P* = 0.41	F_1,24_ = 3.45 *P* = 0.09	–	–	–	–
Body weight	**F**_**1,8**_** = 8.35** ***P* = 0.02**	**F**_**1,8**_** = 11.09** ***P* = 0.01**	**F**_**1,8**_** = 104.63** ***P* < 0.001**	F_1,8_ = 1.84 *P* = 0.21	**F**_**1,8**_** = 7.38** ***P* = 0.03**				

"Handling method × sex" and "handling method × strain" interactions were included in the model. For VIT, handling session (1st, 5th or 9th day), and handling order (before or after handling) were added to the model as explanatory variables and additionally all interactions with the handling method were fitted.

Significant values are in bold.

**Table 2:** Test statistics for the full models run in Experiment 2, where mice were handled briefly twice per week, for five weeks and housed either under CTRL or UCMS conditions. Handling method, strain, sex and housing condition were included as fixed factors and home cage nested in experimenter was included as a random factor.

Experiment 2	Fixed factors										
Outcome measures	Handling method	Strain	Sex	Handling method × strain	Handling method × sex	Before/after handling	Handling session	Handling method × before/after	Handling method × session	Housing condition	Handling method × housing condition
Voluntary interaction with hand	**F**_**1,58**_** = 5.86** ***P* = 0.04**	**F**_**1,56**_** = 30.20** ***P* < 0.001**	**F**_**1,56**_** = 6.62** ***P* = 0.03**	**F**_**1,55**_** = 7.2** ***P* = 0.02**	F_1,56_ = 0.01 *P* = 0.92	**F**_**1,424**_** = 10.01** ***P* = 0.00**3	**F**_**1,475**_** = 20.55** ***P* < 0.001**	F_1,424_ = 2.94 *P* = 0.17	F_1,475_ = 4.27 *P* = 0.08	**F**_**1,56**_** = 8.23** ***P* = 0.01**	F_1,56_ = 0.06 *P* = 0.81
Voluntary interaction with hand after EPM	**F**_**1,66**_** = 16.29** ***P* = 0.0002**	**F**_**1,66**_** = 33.03** ***P* < 0.001**	F_1,66_ = 0.008 *P* = 0.93	**F**_**1,66**_** = 10.54** ***P* = 0.003**	F_1,66_ = 2.39 *P* = 0.25	–	–	–	–	**F**_**1,66**_** = 8.31** ***P* = 0.01**	F_1,66_ = 0.018 *P* = 0.90
Time in EPM open arms	F_1,72_ = 0.21 *P* = 0.65	F_1,72_ = 0.003 *P* = 0.95	F_1,72_ = 0.37 *P* = 0.54	F_1,72_ = 0.45 *P* = 0.50	F_1,72_ = 1.44 *P* = 0.23	–	–	–	–	F_1,72_ = 0.57 *P* = 0.45	**F**_**1,72**_** = 4.71** ***P* = 0.03**
Entries to EPM open arms	F_1,72_ = 3.88 *P* = 0.05	**F**_**1,72**_** = 6.08** ***P* = 0.02**	F_1,72_ = 0.133 *P* = 0.72	F_1,72_ = 1.84 *P* = 0.18	F_1,72_ = 1.69 *P* = 0.20	–	–	–	–	F_1,72_ = 0.32 *P* = 0.57	**F**_**1,72**_** = 6.52** ***P* = 0.01**
Adrenal gland weight	F_1,72_ = 0.09 *P* = 0.77	**F**_**1,72**_** = 94.81** ***P* < 0.001**	**F**_**1,72**_** = 555.10** ***P* < 0.001**	F_1,72_ = 0.0001 *P* = 0.33	F_1,72_ = 0.86 *P* = 0.36	–	–	–	–	F_1,72_ = 1.91 *P* = 0.17	F_1,72_ = 0.066 *P* = 0.80
Sucrose preference	F_1,72_ = 0.52 *P* = 0.47	**F**_**1,72**_** = 9.43** ***P* = 0.003**	F_1,72_ = 0.017 *P* = 0.90	F_1,72_ = 0.032 *P* = 0.86	F_1,72_ = 0.15 *P* = 0.70	–	–	–	–	**F**_**1,72**_** = 32.55** ***P* < 0.001**	F_1,72_ = 0.014 *P* = 0.91
Nest score	F_1,70_ = 1.44 *P* = 0.23	**F**_**1,70**_** = 8.62** ***P* = 0.004**	F_1,70_ = 1.57 *P* = 0.21	F_1,70_ = 2.37 *P* = 0.13	F_1,70_ = 0.22 *P* = 0.64	–	–	–	–	F_1,70_ = 1.89 *P* = 0.13	F_1,70_ = 0.30 *P* = 0.59
Body weight	F_1,72_ = 3.62 *P* = 0.06	F_1,72_ = 1.18 *P* = 0.28	**F**_**1,72**_** = 918.11** ***P* < 0.001**	F_1,72_ = 2.50 *P* = 0.12	F_1,72_ = 0.14 *P* = 0.71					F_1,72_ = 0.18 *P* = 0.67	F_1,72_ = 0.009 *P* = 0.93

	Handling method	Strain	Sex	Handling method × strain	Handling method × sex	Housing condition	Blood collection timepoint	Handling method × blood collection timepoint	Handling method × housing condition	Blood collection timepoint × housing condition	
Plasma corticosterone levels	F_1,72_ = 0.31 *P* = 0.58	**F**_**1,72**_** = 86.22** ***P* < 0.001**	**F**_**1,72**_** = 22.77** ***P* < 0.001**	F_1,72_ = 0.13 *P* = 0.71	F_1,72_ = 009 *P* = 0.92	F_1,72_ = 3.88 *P* = 0.06	**F**_**1,391**_** = 506.92** ***P* < 0.001**	F_2,391_ = 0.65 *P* = 0.53	F_1,72_ = 0.122 *P* = 0.73	**F**_**1,391**_** = 3.11** ***P* = 0.04**	

"Handling method × sex", "handling method × strain" and "handling method × housing condition" interactions were included to the model. For VIT, handling session (1st or 2nd), and handling order (before or after handling) were added to the model as explanatory variables and additionally all interactions with the handling method were fitted. For plasma corticosterone analysis, blood collection timepoint (initial, reaction, and recovery) and interaction with housing condition and handling method was added to the model.

Significant values are in bold.

#### Voluntary interaction test (VIT)

**Figure 3:**
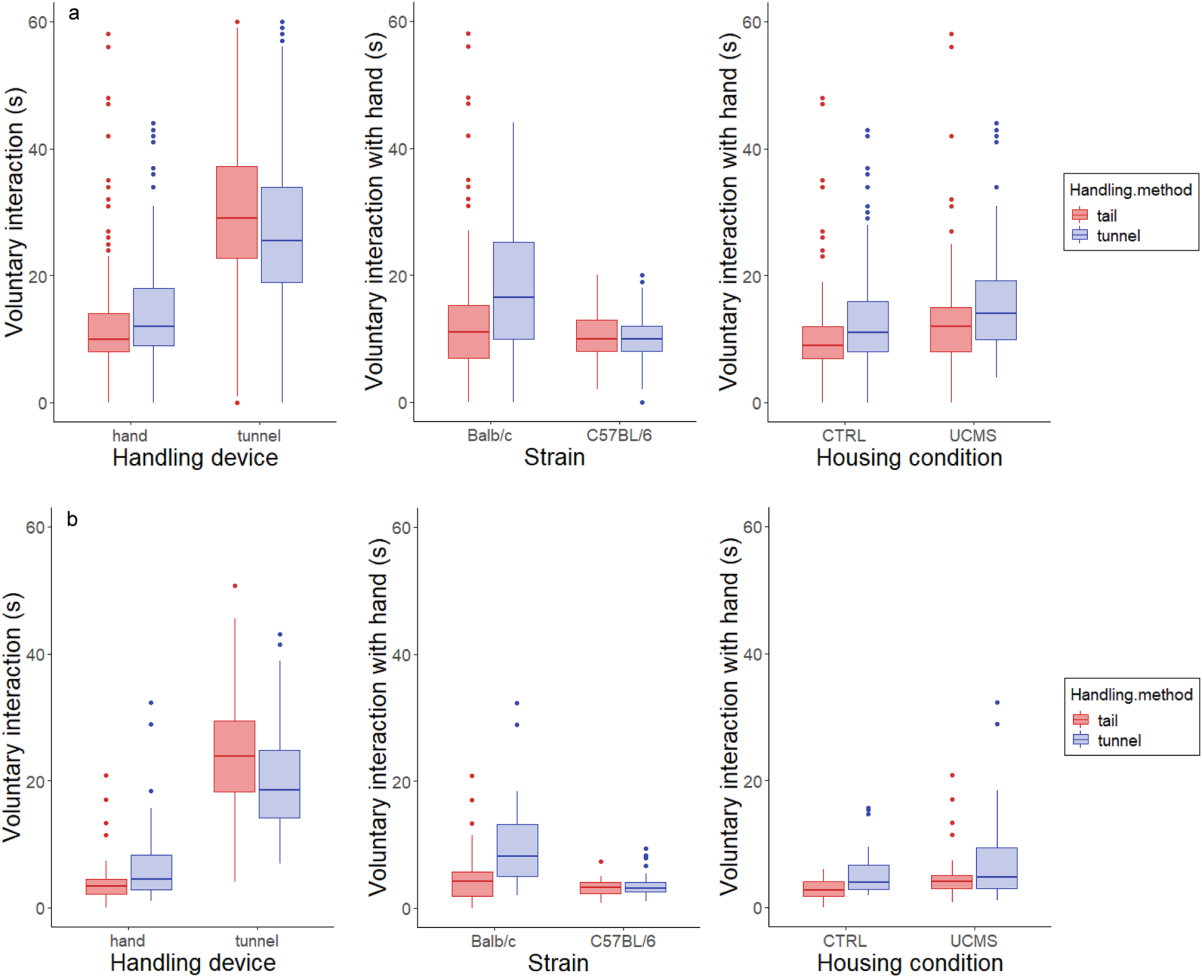
Voluntary interaction with both handling devices, experimenter's hand and tunnel, when mice were briefly handled twice per week. (**a**) Voluntary interaction with the hand at weeks two and four of housing period and (**b**) after EPM testing. Both during housing and after EPM testing, mice interacted with the tunnel more than with the experimenter's hand. Handling method affected voluntary interaction with the experimenter's hand in Balb/c mice. UCMS housed mice also interacted with the experimenter slightly more than CTRL housed mice. Here, both interaction before and after handling are included.

Voluntary interaction was measured twice during housing (at week 2 and 4) and immediately after EPM testing. Time spent interacting was influenced by handling method and handling device (handling method × handling device: F_1,928_ = 26.58, *P* < 0.001, Fig. 3a). We only looked at interaction with the hand for subsequent analyses. Handling method affected time spent interacting with the experimenter's hand in Balb/c mice, where tunnel handled mice interacted longer than tail handled mice (handling method × strain interaction: F_1,55_ = 7.22, *P* = 0.02, 18 ± 1 s compared to 13 ± 1 s). Interaction with the hand also slightly increased across the two handling sessions from 11 ± 0.5 to 14 ± 0.5 s (F_1,475_ = 20.55, *P* < 0.001, Figure S2a) and immediately after the handling session compared to before (from 12 ± 0.4 to 14 ± 0.7 s; F_1,424_ = 10.10, *P* = 0.003). Interestingly, UCMS housed mice interacted with the hand slightly more than CTRL housed mice (15 ± 0.6 compared to 12 ± 0.5 s; F_1,56_ = 8.23, *P* = 0.01, Fig. 3a). Males also interacted more than females (14 ± 0.6 to 12 ± 0.5 s; F_1,56_ = 6.62, *P* = 0.03, Figure S2a).

Voluntary interaction was also measured immediately after EPM testing, where we found a similar pattern. Interaction was influenced by handling method and handling device (handling method × handling device; F_1,232_ = 24.62, *P* < 0.001, Fig. 3b). When we looked at voluntary interaction with the experimenter's hand only, we found that only in the Balb/c strain, tunnel handled mice interacted with the hand more (handling method × strain; F_1,66_ = 10.54, *P* = 0.003, 10 ± 1 s compared to 5 ± 1 s), whereas interaction time for C57BL/6 mice was similar with both handling methods (3 ± 0.5 s). There was also an overall effect of housing on the time spent interacting with the handler's hand (F_1,66_ = 8.31, *P* = 0.01, Fig. 3b), whereby mice exposed to UCMS interacted more with the experimenter's hand than mice housed under CTRL conditions (6 ± 0.6 compared to 4 ± 0.4 s), however the effect size was very small.

#### Elevated plus-maze (EPM)

**Figure 4:**
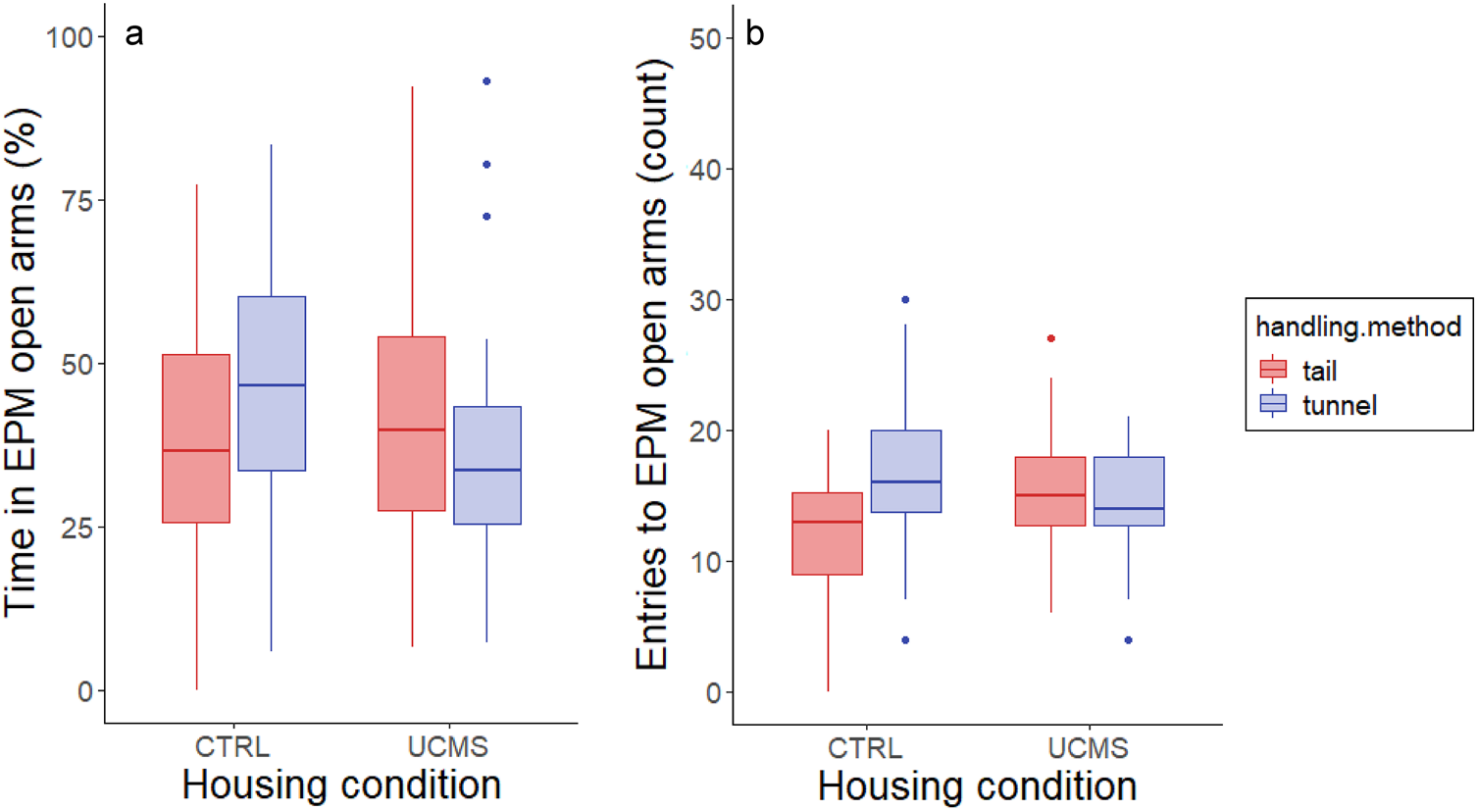
Behaviour on the elevated plus-maze. (**a**) Time spent and (**b**) number of entries to the open arms of the EPM. Handling method affected time spent in open arms and open arm entries of the EPM in CTRL housed mice.

We found an effect of handling method × housing condition on time spent (F_1,72_ = 4.71, *P* = 0.03) and entries to open arms of the EPM (F_1,72_ = 6.52, *P* = 0.01). Tunnel handled CTRL housed mice spent more time in open arms (46 ± 3%) and made more entries to open arms (16 ± 1) compared to tail handled mice (37 ± 3% and 12 ± 1; Fig. 4a and b). Balb/c mice made fewer open arm entries compared to C57BL/6 mice (F_1,72_ = 6.08, *P* = 0.02; Figure S3b) but time spent on open arms was not different (Table 2, Figure S3a). Also sex had no effect on time on open arms or open arm entries (Table 2, Figure S3). Neither handling method (F_1,72_ = 0.16, *P* = 0.69) nor housing condition (F_1,72_ = 0.72, *P* = 0.40) affected total distance travelled in the EPM.

#### HPA axis reactivity test

**Figure 5:**
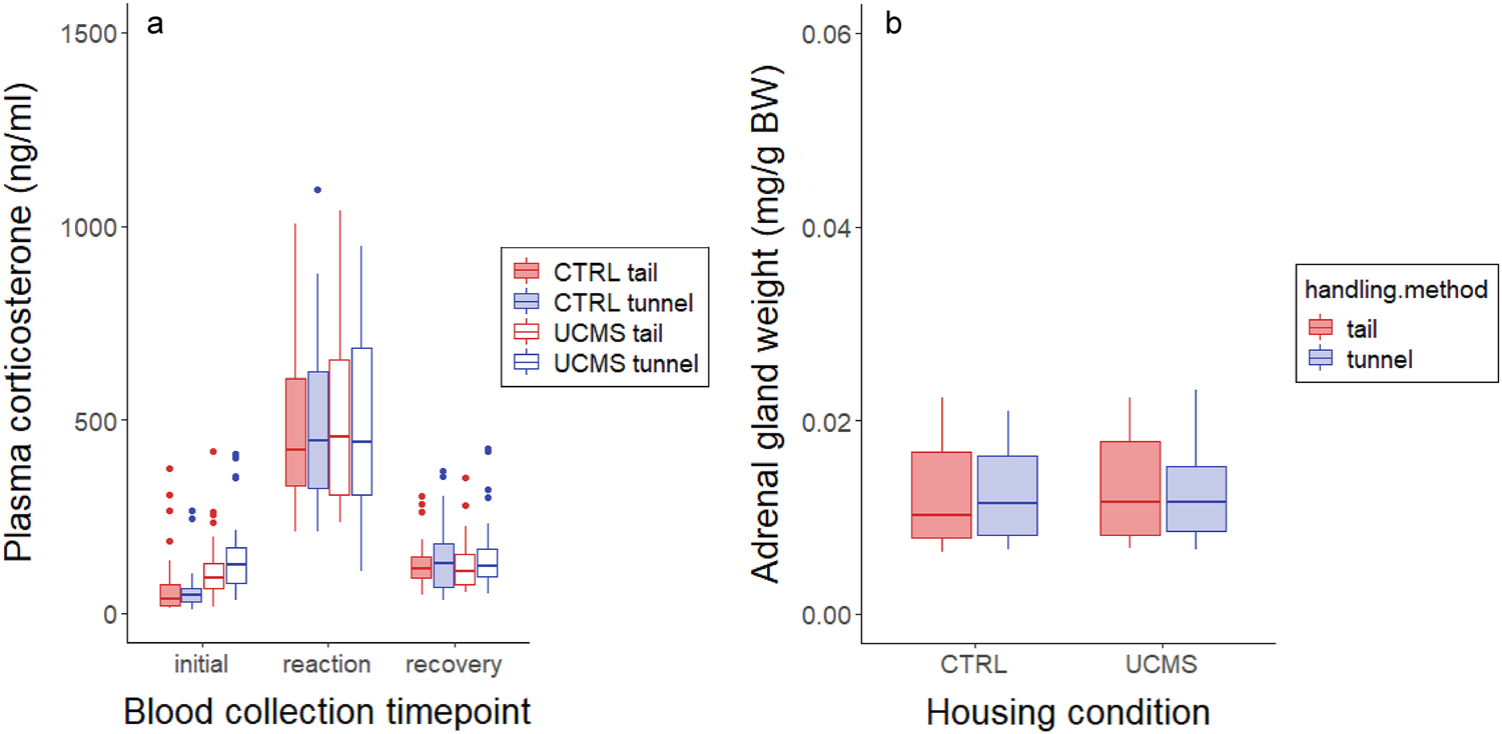
(**a**) Plasma corticosterone levels before 20 min restraint (initial value), immediately after restraint (reaction) and 70 min after restraint (recovery). UCMS housed mice had higher initial plasma corticosterone levels. There was no difference in corticosterone levels between tunnel and tail handled mice. (**b**) Adrenal gland weights relative to body weight did not differ between UCMS and CTRL mice nor between tail and tunnel handled mice.

Housing condition influenced initial plasma corticosterone levels (housing × blood collection timepoint interaction; F_2,391_ = 3.11, *P* = 0.04). Thus, initial corticosterone levels of mice housed under UCMS conditions were twice as high as those of CTRL mice (126 ± 10 compared to 63 ± 8 ng/ml; post hoc comparison F_1,72_ = 15.01, *P* = 0.001; Fig. 5a). There was no effect of UCMS housing on corticosterone levels during the acute stress response (F_1,72_ = 0.006, *P* = 0.94) or recovery (F_1,72_ = 0.03, *P* = 0.87). There was also no difference in plasma corticosterone levels between the two handling methods (F_1,469_ = 0.31, *P* = 0.58). Overall plasma corticosterone levels were higher in the Balb/c strain (F_1,72_ = 86.22, P < 0.001) and in females (F_1,72_ = 22.77, P < 0.001; Figure S4a).

#### Adrenal gland weights

Adrenal gland weights did not differ between tail and tunnel handled mice (F_1,72_ = 0.09, *P* = 0.77), nor between UCMS and CTRL mice (F_1,72_ = 1.91, *P* = 0.17; Fig. 5b). Adrenal glands were heavier in Balb/c mice (F_1,72_ = 94.81, *P* < 0.001) and in females (F_1,72_ = 555.1, *P* < 0.001; figure S4b). There was no effect of housing condition (F_1,72_ = 0.18, *P* = 0.67) or handling method (F_1,72_ = 3.62, *P* = 0.06) on body weight at euthanasia.

#### Sucrose preference

**Figure 6:**
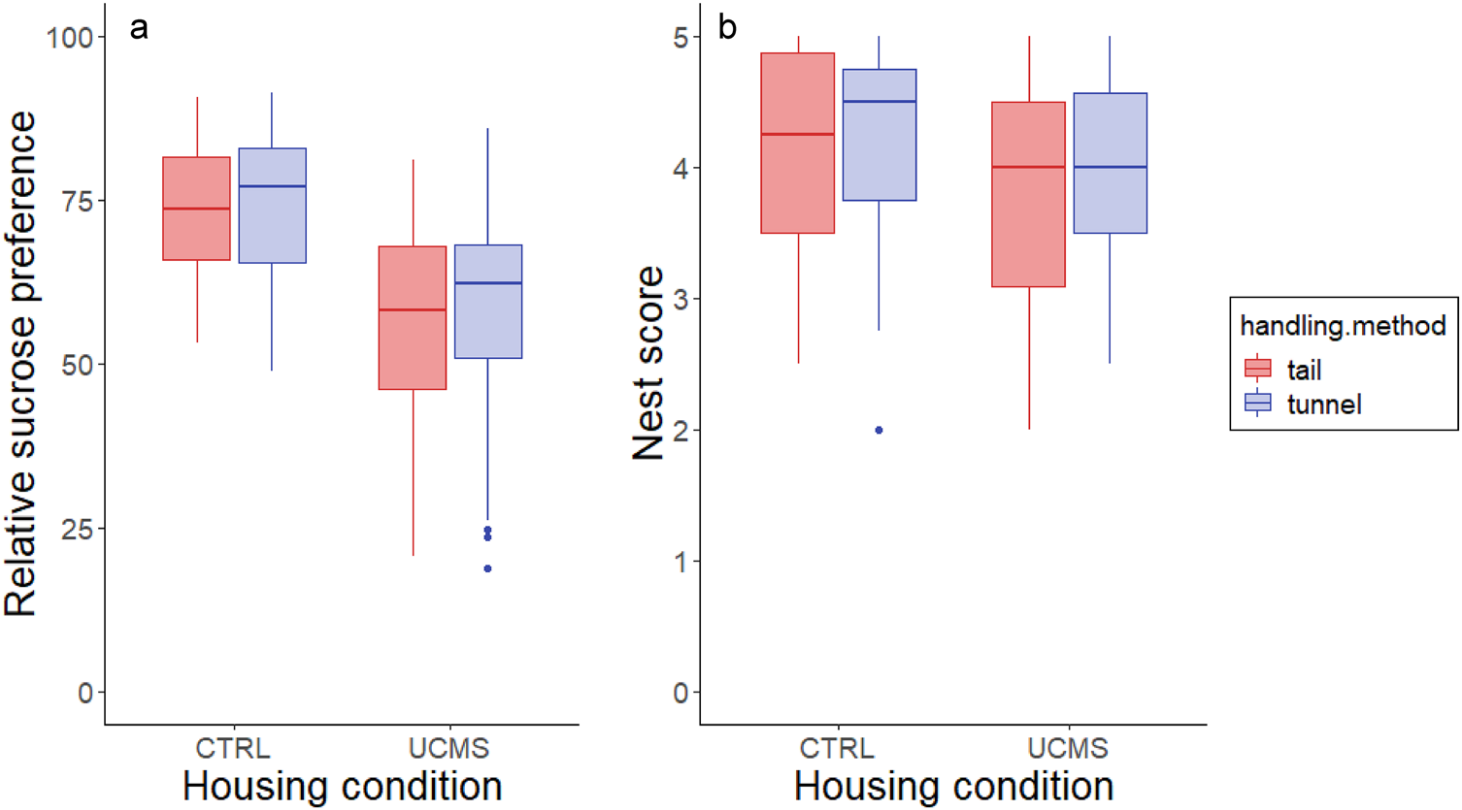
(**a**) Relative sucrose preference was lower in UCMS mice. Handling method had no effect on relative sucrose preference. (**b**) Nest scores did not differ between UCMS and CTRL mice nor between tail and tunnel handled mice.

UCMS housed mice had a lower preference for sucrose (57 ± 2% compared to 74 ± 1% in CTRL mice; F_1,72_ = 32.55, *P* < 0.001; Fig. 6a), but there was no difference in preference between differentially handled mice (F_1,72_ = 0.52, *P* = 0.47). Sucrose preference was lower in Balb/c mice (F_1,72_ = 9.43, *P* = 0.003; Figure S5a), while sex had no effect (Table 2).

#### Nest score

There was no difference in the quality of nests between tail and tunnel handled mice (F_1,70_ = 1.44, *P* = 0.23) or between differentially housed mice (F_1,70_ = 1.89, *P* = 0.13; Fig. 6b). Balb/c mice made lower quality nests compared to C57BL/6 mice (F_1,70_ = 8.62, *P* = 0.004; Figure S5b), while sex had no effect (Table 2).

## Discussion

Our data suggest that both extensive daily tail handling, as well as minimal routine tail handling of laboratory mice can increase behavioural measures of anxiety and avoidance of the handler’s hand, and some of these effects are strain–dependent. However, routine tail handling twice per week did not activate the HPA axis or induce behavioural changes similar to those seen under unpredictable chronic mild stress^33–35,37^.

In Experiment 1, we show that in Balb/c mice, daily tail handling decreased open arm exploration in the EPM. However, we did not find differences in voluntary interaction with the experimenter between daily tail and tunnel handled mice as reported in other studies^11–14^. This could be due to a relatively small sample size, since several VIT trials had to be interrupted due to aggression towards the cage mate or the experimenter's hand. Our assessment of voluntary interaction also differed from previous studies, as we measured the animals' interaction with both the experimenter's hand and the handling tunnel for all mice. Most previous studies have measured time interacting with the handling device, which in tail handled mice is the experimenter's hand and in tunnel handled mice the tunnel^12–14,17,20,38^ (but see^15,39^). However, mice show strong thigmotaxis^40^, especially under white light conditions^41^, and the red tunnel may serve as a dark shelter, which may increase their preference for the tunnel, regardless of the handling method. Our data confirm this, as overall, mice spent more time interacting with the tunnel compared to the experimenter's hand. This is further confirmed in Experiment 2 where tail handled mice interacted with the tunnel more than tunnel handled mice, further suggesting that tail handled mice may have been using the tunnel as a shelter, hiding after being disturbed. Therefore, to avoid potential confounds, we used voluntary interaction with the experimenter's hand as a measure of habituation to the handler and found that when handled daily, voluntary interaction increased across handling sessions, suggesting that mice gradually habituated to the experimenter.

We found a similar effect of the handling method on behavioural measures of anxiety in Experiment 2, when mice were handled during husbandry twice per week, for five weeks, mimicking routine handling that mice experience in breeding and research facilities. Thus, tail handled mice housed under standard laboratory (CTRL) conditions showed slightly reduced exploration of the open arms in the EPM. Tail handled Balb/c mice also spent less time interacting with the handler compared to tunnel handled mice, while in C57BL/6 mice interaction with the handler was unaffected by the handling method. Mouse strains differ in susceptibility to environmental stressors, with Balb/c mice exhibiting elevated levels of behavioural measures of anxiety and stress reactivity compared to the relatively stress-resilient C57BL/6 mice^28,42–45^. While strain-dependent effects of handling methods on behavioural measures of anxiety have not been extensively investigated, our results are consistent with strain-specific effects of handling method on open field activity in Balb/c mice^22^. Therefore, in view of refinement, strains of mice may differ in their sensitivity to handling stress.

While less aversive handling (tunnel and cup handling) was found to reduce behavioural measures of anxiety in some strains of mice^11,13^, evidence for an effect of handling method on other measures of stress is more ambiguous^14,16,46,47^. To determine whether tail handling represents a source of chronic stress, we compared routine tail handling with UCMS, a validated procedure to induce chronic mild stress^24^. Five weeks of housing under UCMS affected HPA axis activity and induced anhedonia, two measures that are commonly affected by chronic stress^31,37,48^. Sucrose preference of UCMS housed mice was below 65%, which is a commonly accepted criterion for anhedonia^49,50^, as it correlates with other measures of impaired welfare (increased threshold for intracranial self-stimulation^51^, decreased latency and increased duration of REM sleep^52^, and alterations of circadian rhythms^53^). Anhedonia can also be used as a proxy measure of affective state, as reduced sucrose preference can be restored by antidepressants^54,55^. However, UCMS did not affect HPA axis reactivity to an acute stressor (restraint), nest quality, behavioural measures of anxiety, and adrenal gland weight. Sustained activation of the HPA axis during chronic stress leads to elevated basal corticosterone levels and adrenal gland hypertrophy^32,56^, but this effect may only appear after longer exposure to UCMS^57^. While nest quality may be a promising cage-side assessment tool for recognizing distress^58,59^, it appears to be more sensitive to changes in pain or sickness behaviour^58,60^, than chronic stress. Whether UCMS induces anhedonia and behavioural measures of despair without triggering measures of anxiety (as measured in the EPM) has remained elusive^25,27^.

Handling method also did not affect chronic stress markers. Similar findings were reported by Miller et al. who found no detectable difference in cancer symptoms between differentially handled mice^46^. In fact, only a few studies report chronic effects of handling method on stress markers, but these seem to be sex dependent^14,16^ or specific to pregnant dams^47^. Our results also contradict those reported by Clarkson et al.^14^, who found tail handled mice to be more anhedonic. However, their measure of anhedonia (absolute sucrose intake) differs from measures that are commonly used in the stress literature, such as sucrose intake relative to body weight or sucrose preference, which are more reliable measures of the animal's response to a sweet solution^61^.

While our results do not corroborate the observation of tail handling being a source of chronic stress as reported in some other studies^14,16^, we also found no effect of UCMS on behavioural measures of anxiety, indicating that routine handling and UCMS may be perceived differently by mice. While UCMS protocols often include a variety of stressors, handling is limited to one type of stressor that likely became predictable throughout the study. Responses to different stressors can be complex, and predictable stress (such as repeated restraint) has been shown to have a less negative impact on immobility and anhedonia compared to UCMS, as animals habituate to repeated exposure to restraint^29,62^. Habituation to handling and the experimenter was also evident in our study as both daily and routine handled mice increased voluntary interaction with the experimenter's hand across handling sessions independent of the handling method, which was also reported by López-Salesansky et al.^63^. It is also possible that behavioural changes in the EPM and VIT may be a more acute response to handling. Similar effects were reported by Ueno et al.^64^, who showed that mice exposed to tool-free handling showed less avoidance of the open arms in the EPM compared to handled mice, however, no difference was found in immobility in the forced swim test.

Based on our results, we cannot firmly conclude how different handling methods are perceived by the animals and further work is needed to infer the influence handling has on the animals' affective state. Both animal welfare and data quality should be the main drivers to justify refinements of husbandry procedures. Given that even routine handling can alter behavioural measures of anxiety, thereby potentially affecting readouts of animal experiments^12,13^, the promotion of less aversive handling methods appears justified, especially in more sensitive strains of mice. Our results also further underline the need to appreciate strain differences in responses to routine husbandry procedures.

## Materials and methods

This study was carried out in accordance with guidelines of the Swiss Animal Welfare Ordinance (TschV 455.1). It was approved by the Cantonal Veterinary Office in Bern, Switzerland (permit number BE8/20). The reporting of the study follows the ARRIVE guidelines for reporting animal research^65^.

### Animal housing and care

For this study, we used 192 mice from two strains (C57BL/6JRj and Balb/cRj, referred to as C57BL/6 and Balb/c throughout the manuscript) and of both sexes, of which 32 (8 per strain and sex) were used in Experiment 1 and 160 (40 per strain and sex) were used in Experiment 2. Power was calculated using G-power, version 3.1.9.2, for a minimal power of 0.8 and alpha set to 0.05. The primary outcome variable in Experiment 1 was the means difference of "time in open arms" in the EPM. Estimates for expected effects were taken from Clarkson et al.^14^. The primary outcome in Experiment 2 was plasma corticosterone concentration. Corticosterone values for the power analysis for tail vs tunnel handling were taken from Ghosal et al.^17^. We expected UCMS females to have 60% higher corticosterone levels than males (based on Furman et al.^66^), Balb/c having 15% higher levels than C57BL/6 (based on Laber et al.^67^) and UCMS animals having 60% higher corticosterone levels than non-stressed animals (based on Furman et al.^66^). The experimental design is a fully crossed 2 × 2 × 2 × 2 design, with strain, sex, stress housing and handling method as binary factors. The experiment was powered to allow detecting first-order interactions between handling method and housing with a power of 0.8 (alpha-level set to 0.05). All mice were purchased from Janvier Laboratories, France at the age of seven (Experiment 1) and three weeks (Experiment 2), respectively.

All animals were housed on a 12:12 h light–dark cycle with lights off at 21 h. Temperature in both housing rooms ranged from 21 to 23 °C and relative humidity was between 40–56%. Animals were housed in same-sex and same-strain pairs in Type 3 cages (Tecniplast, 425 × 276 × 153 mm), with woodchip bedding (SAFE®), approximately 10 g nesting material (Sizzle nest, Datesand), a red tunnel (Datesand) and a red mouse house (Tecniplast), with food (Kliba Nafag, 3430) and tap water provided ad libitum*.* Cages were changed once per week in the housing room. Balb/c mice were marked for identification using black fur marker (Stoelting) and C57BL/6J mice were marked with a fur cut above the tail.

### Experiment 1: Daily handling for nine consecutive days

**Figure 7:**
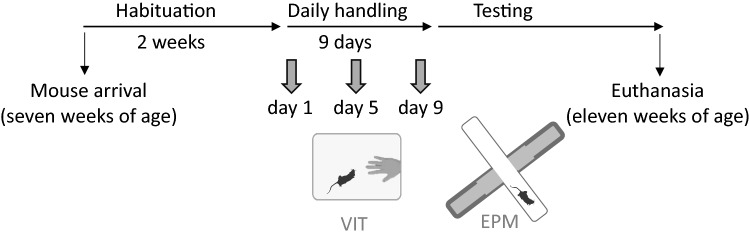
Timeline of Experiment 1. After habituation to the facility, the animals were handled daily for nine days. Animals were tested for voluntary interaction (VIT) three times during the handling period, and thereafter in an elevated plus-maze (EPM) test once. Mouse image reprinted from Scidraw doi.org/10.5281/zenodo.3925917.

Cages were randomly (random.org) allocated to one of the handling groups (tail or tunnel) and one of the experimenters (JN or IJ), who handled the same animals throughout the study. Sexes and strains were equally split between the two handling methods and experimenters. Cages were distributed on one cage rack in a balanced order. Mice were handled by their designated method by the same handler throughout the housing and experimental period. Handling began when mice were nine weeks old, two weeks after arrival to the facility, during which time they were left undisturbed, apart from weekly cage changes. Handling sessions were performed once per day, for nine days (Fig. 7), to replicate effects of tail handling method using the original daily handling protocol^11,12^. Handling sessions started half an hour after the onset of the light phase and lasted approximately 2 h. Mice were handled using the two methods described by Hurst and West^13^. Briefly, mice handled by the standard tail handling method were held by the base of their tail and lifted onto the palm of the other hand where they were held by the tail for 30 s before being released back into the cage. Tunnel handled mice were picked up using a home cage tunnel and lifted above the cage for 30 s, while remaining in the tunnel. Animals received two 30 s handling sessions per day, 60 s apart. Both experimenters wore nitril gloves, which were changed between cages.

#### Voluntary interaction test (VIT)

Mice were tested for voluntary interaction with the experimenter as previously described by Hurst and West^13^. Voluntary interaction was measured for 60 s immediately before and after each handling session. VIT was conducted on days 1, 5 and 9 during the handling period and started half an hour after lights on. For all tests, nesting material and other enrichments were removed from the cage.

Tail handled mice were first tested for interaction with the hand followed by the hand holding the tunnel, while for tunnel handled mice the order was reversed. VIT sessions were recorded by an overhead video camera (Videocomponents, Germany) for later analysis. Time interacting with the experimenter's hand or tunnel was recorded^12,13^. For video analysis, all videos were cut so that the coder (JN) was blind to the handling method and the time when the voluntary interaction was measured (before or after handling).

#### Elevated plus-maze (EPM) test

After nine handling sessions animals were tested for anxiety-related behaviour in the elevated plus-maze (EPM) test. The apparatus was elevated 40 cm above floor level and was made of gray polycarbonate with infrared (850 nm) backlit floors. It consisted of four arms, each 30 cm in length and 6 cm wide, and a center square measuring 6 × 6 cm. Two arms opposite to each other were open, with a small lip around the perimeter 0.5 cm high, while the remaining two arms were enclosed, with walls 15 cm high. Mice were tested in a test room adjacent to the housing rooms, between the 1st and 5th hour of the light phase. Light intensity in the test room during testing was 120 lx. Each test started by taking the mouse from the home cage and placing it in the center part of the EPM, using the designated handling method and facing the closed arm. The mouse was left to explore the EPM for 5 min. Both animals from the cage were tested at the same time by their respective handlers, using two identical apparatuses placed next to each other but visually separated. Test order was balanced across handling method and strain. Males were tested first, followed by females on the same day. Trials were analysed by automated video tracking software (Noldus Ethovision XT 9.0). Criterion for arm entry was when center-point of the animal (as detected by Ethovision) was in the arm. Time spent in open arms (relative to time spent in all four arms, excluding the central zone) and entries to open arms were used for analysis. Between trials, the apparatuses were cleaned with detergent and water.

#### Thymus and adrenal weights

After EPM testing, mice were euthanized with Isoflurane anaesthesia, followed by CO_2_ inhalation and decapitation. Thymus and adrenal glands from all animals were removed, dissected from fat, and weighed on an analytical scale (Mettler AE160, Mettler-Toledo, Switzerland).

### Experiment 2: Weekly handling and UCMS

After arrival to the facility, cages were randomly (random.org) assigned to one of four treatment groups using a 2 × 2 factorial design, with the two factors housing conditions (unpredictable chronic mild stress (UCMS) or control housing conditions (CTRL)) and handling (tail handling or tunnel handling). Cages were also randomly assigned to one of the experimenters (JN or IJ) who handled the same animals with their designated method throughout the study. Sexes and strains were equally split between the two handling methods. Cages were distributed on cage racks in a balanced order, with males and females housed on separate racks in the same housing room. CTRL and UCMS mice were housed in separate rooms. All animals were handled twice per week, once for a routine health check and once during the weekly cage change. Handling also differed in duration from the procedure applied in Experiment 1. Here, the animal was picked up by the tail or the home cage tunnel, lifted onto the back of experimenter's hand and held there for approximately 2 s. Tunnel handled mice remained in the tunnel and were visually checked through the transparent tunnel. Animals were then transferred either back in the home cage (during health checks) or to a weighing cage and then using the same handling method, in a new cage (during cage change). Handling duration therefore did not exceed a couple of seconds and more closely mirrored standard husbandry procedures.

**Figure 8:**
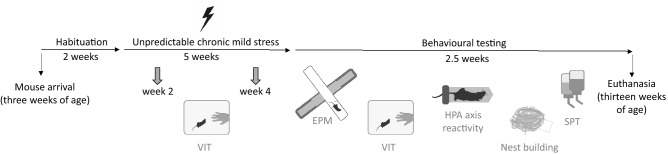
Timeline of Experiment 2. After habituation to the facility, the animals were handled twice weekly throughout the housing and testing period. Animals were tested for voluntary interaction (VIT) twice during the housing period and once after the elevated plus-maze (EPM) testing. In addition, animals were tested in the EPM, hypothalamic–pituitary–adrenal (HPA) axis reactivity after physical restraint, nest building and for anhedonia using a sucrose preference test (SPT). Mouse image reprinted from Scidraw doi.org/10.5281/zenodo.3925917.

CTRL mice were housed under a constant 12:12 h light:dark cycle while UCMS mice were exposed to several stressors of variable duration in an unpredictable order over the 5 week housing period (from five to ten weeks of age, Fig. 8) to put the effects of handling method in perspective with chronic mild stress. The following stressors were included in the UCMS condition (Table 3): home cage tilted backwards by 45 deg (tilted cage), lights on for an entire 24 h cycle (lights on), all enrichment removed for 24 h (barren cage), 600 ml of water added to bedding material during the dark phase (wet bedding), alternating 30 min periods of light and dark (alternation of dark and light cycle), placing mice in a cage previously occupied by other mice of the same sex and strain (social stress), exposure to predatory noises from a natural predator (meowing cat) for 4 h (predatory noise). Stressors were applied in both dark and light phase. Stressors that required transferring the animals to a new cage (transfer from wet bedding and social stress) were applied so that the transfer to a new cage coincided with the regular weekly cage change. This was to ensure that both CTRL and UCMS mice were handled with the same frequency throughout the experiment.

**Table 3:** The order in which stressors were presented during the UCMS.

Week	Day	Stressor	Duration
One	Monday	Wet bedding	21 h–10 h
	Tuesday	Tilted cage	21 h–09 h
	Wednesday	Alternation light:dark cycle	11 h–21 h
	Thursday	Social stress	10:30 h–13:30 h
	Friday	Barren cage	22 h–09 h
	Saturday	Predatory noise	09:40 h–13:40 h
	Sunday	Lights on	09 h–09 h
Two	Monday	Predatory noise	10:30 h–14:30 h
	Tuesday	Social stress	11 h–16 h
	Wednesday	Wet bedding	22 h–10:30 h
	Thursday	Barren cage	11 h–21 h
	Friday	Lights on	09 h–09 h
	Saturday	Alternation light:dark cycle	21 h–09 h
	Sunday	Tilted cage	9 h–21 h
Three	Monday	Wet bedding	21 h–09 h
	Tuesday	Barren cage	21 h–09 h
	Wednesday	Predatory noise	21 h–01 h
	Thursday	Social stress	10 h–15 h
	Friday	Tilted cage	21 h–09 h
	Saturday	Lights on	09 h–09 h
	Sunday	Alternation light:dark cycle	09 h–21 h
Four	Monday	Barren cage	09 h–21 h
	Tuesday	Social stress	9 h–14 h
	Wednesday	Wet bedding	21 h–09 h
	Thursday	Predatory noise	22 h–02 h
	Friday	Tilted cage	10 h–21 h
	Saturday	Lights on	09 h–09 h
	Sunday	Alternation light:dark cycle	21 h–9 h
Five	Monday	Barren cage	15 h–9 h
	Tuesday	Wet bedding	7:30 h–14 h
	Wednesday	Alternation light:dark cycle	12 h–22 h
	Thursday	Social stress	09 h–15 h
	Friday	Lights on	09 h:09 h
	Saturday	Tilted cage	10 h–22 h

#### Voluntary interaction test (VIT)

VIT was measured immediately before and after the handling session in week 2 and 4 of the housing period, as well as immediately after the transfer of the mice from the EPM back into the home cage (Fig. 8). The procedure was the same as described in Experiment 1. Because of the large number of animals and since we were mainly interested in the comparison of voluntary interaction between tail handled and tunnel handled mice, VIT for CTRL and UCMS animals was done on separate days.

#### Elevated plus-maze (EPM) test

Two days after the UCMS period had ended, we started testing the mice for commonly used behavioural measures of anxiety in the EPM. Mice were tested across four consecutive days, with males and females being tested on alternate days. Within each test day, the test order was balanced for handling method, experimenter, housing condition and mouse strain. Testing was done in the light phase and the procedure was the same as in Experiment 1.

#### HPA axis reactivity test

Chronic stress can alter HPA axis activity and reactivity to acute stressors^36,68^. Mice underwent a stress reactivity test as described by Touma et al.^48^. Briefly, mice had blood samples collected from the tail vessel using a minimal-invasive sampling method^69^ to determine initial levels of plasma corticosterone, followed by a 20 min physical restraint in a 50 ml plastic conical tube (11.5 cm × 2.5 cm, Fisher Scientific AG, Reinach, Switzerland) with custom made holes (for breathing and tail). The second blood sample was collected immediately after removal from the restraint tube (reaction levels). The animals were returned to the home cage and left undisturbed for 70 min, when the third blood sample (recovery) was collected. This procedure allowed us to assess plasma corticosterone levels at different stages of the HPA axis response to an acute stressor. Blood was collected from a small incision in the ventral tail vessel with a scalpel (Paragon disposable scalpels No.10, Paragon Medical Lausanne, Switzerland) and for each blood collection a new incision rostral to the previous one was made. Mice were sampled across four consecutive days, with males and females being tested on alternate days. Within each test day, the test order was balanced for handling method, experimenter, housing condition and mouse strain. Both cage mates were sampled at the same time by two experimenters (IJ and RR). Blood samples were collected using a dipotassium-EDTA capillary blood collection system (Microvette® CB 300 K2E, Sarstedt, Nümbrecht, Germany). Immediately after sampling, the blood samples were placed on ice and centrifuged for 10 min at 4000 g and 4 °C. Plasma was stored at − 80 °C and sent to University of Osnabrück where it was analysed for corticosterone concentrations as described by Touma et al.^48^. Intra-assay coefficient of variation for plasma corticosterone measure was 10,9% and Inter-assay coefficient of variation was 15,2%.

#### Sucrose preference test (SPT)

Sucrose preference was assessed across three days. Animals were first habituated to 1% sucrose solution by placing a drinking bottle into a home cage for 24 h. Animals were then singly housed for three consecutive days in Type 3 cages with nesting material (Sizzle nest, Datesand), a red tunnel (Datesand) and a red house (Tecniplast). Food and water were available ad libitum and a bottle of 1% sucrose solution was placed in each cage. The position of the water and sucrose bottles was changed daily, to avoid side preference. Both bottles were weighed daily to determine water and sucrose consumption in g per day. Daily sucrose preference was calculated by percentage of sucrose consumed (measured in g) relative to all liquid (sucrose solution + water) consumed. Average preference was calculated across the three test days.

#### Nest score

Cage assessments, such as nest quality can be used as an indicator of wellbeing^58,60,69,70^, as laboratory mice are highly motivated to build nests when provided with appropriate nesting material^58^. Nests were scored for each mouse, after single housing for the sucrose preference testing, according to the protocol adapted from Gaskill et al.^58^. Nest quality was assessed 24 h after provision of 10 g fresh shredded paper and one nestlet. All cages were re-labelled (by MR) and scored independently by two observers (JN and IJ) blind to the cage ID. Briefly, a score between 1 and 5 was assigned depending on the shape of the nest. For each mouse, the average nest score from both observers was used for analysis.

#### Adrenal weights

After SP testing, mice were euthanized with Isoflurane anaesthesia, followed by CO_2_ inhalation and decapitation. Adrenal glands were collected postmortem from all animals and weighed on analytical scale (Mettler AE160, Mettler-Toledo, Switzerland).

### Statistical analysis

Statistical analyses were carried out using R 3.6.0^71^. For each model, we checked the distribution of residuals using frequency histograms and normal Q-Q plots to assess any major deviation from normality. Linear mixed effects models (*lmer4* package) were used to assess effects of handling method and UCMS treatment on behavioural and physiological measures.

#### **Experiment 1**

We ran full mixed models for all outcome measures, with handling method (tail vs tunnel), strain and sex included as fixed factors and home cage nested in experimenter was included as a random factor. Handling method × sex and handling method × strain interactions were included to the model. For VIT, handling device (hand vs tunnel), handling session (1st, 5th or 9th day), and handling order (before or after handling) were added to the above model as explanatory variables. All interactions with the handling method were fitted.

#### **Experiment 2**

In Experiment 2, where mice were housed either under CTRL or UCMS conditions, housing condition was included as a fixed factor to the above full model, and the handling method x housing condition interaction was added to the model. Nest scores had a negatively skewed distribution, therefore, the data were reversed and analysed with negative binomial mixed model.

## Supplementary Information

Supplementary Information 1.

Supplementary Information 2.

Supplementary Information 3.

Supplementary Information 4.

Supplementary Information 5.

Supplementary Information 6.

## Data Availability

All data is available at the BORIS repository (https://doi.org/10.48620/166).
