# Supplementary material for: Handling method affects measures of anxiety, but not chronic stress in mice
Source: Sci Rep. 2022 Dec 3;12:20938. doi: 10.1038/s41598-022-25090-9 (PMC9719500; doi:10.1038/s41598-022-25090-9)
Supplement: Supplementary file 1 — Supplementary Information 1. [file 41598_2022_25090_MOESM1_ESM.docx]

**Handling method affects measures of anxiety, but not chronic stress in mice**

Janja Novak^1*^, Ivana Jaric^1^, Marianna Rosso^1^, Reto Rufener^2^, Chadi Touma^3^, Hanno Würbel^1^

**Supplementary Figures**

**Figure S1.** (a.) Adrenal gland and (b.) thymus weights in Experiment 1, when animals were handled daily. Females had heavier adrenal glands and thymus. C57BL/6 mice also had heavier thymus.

**Figure S2.** Voluntary interaction with the hand (a.) during housing and (b.) after EPM testing in Experiment 2, when mice were briefly handled twice per week. Interaction with experimenter's hand increased across the two handling sessions during housing. Males also interacted more than females during housing but not after EPM testing.

**Figure S3.** (a.) Time spent in EPM open arms and (b.) number of EPM open arm entries per strain and sex in Experiment 2, when mice were briefly handled twice per week. Time in EPM open arms did not differ between the two strains or sexes. Balb/c mice made fewer entries to open arms compared to C57BL/6mice.

**Figure S4.** (a.) Plasma corticosterone levels and (b.) adrenal gland weights per strain and sex. Overall plasma corticosterone levels and adrenal gland weights were higher in Balb/c strain and in females.

**Figure S5.** (a.) Relative sucrose preference and (b.) nest score per strain and sex. Balb/c mice had lower sucrose preference and nest score. There was no difference in these two measures between the two sexes.
